# Comparative Antioxidant Activity and Total Flavonoid Content of Persian Pomegranate (*Punica granatum L.*) Cultivars

**Published:** 2011

**Authors:** Mohammad Reza Shams Ardekani, Mannan Hajimahmoodi, Mohammad Reza Oveisi, Naficeh Sadeghi, Behrooz Jannat, Ali Mohammad Ranjbar, Narges Gholam, Tahereh Moridi

**Affiliations:** a Department of Traditional Pharmacy, School of Traditional Iranian Medicine, Tehran University of Medical Sciences, Tehran, Iran.; b Drug and Food Control Department, Faculty of Pharmacy, Tehran University of Medical Sciences, Tehran, Iran.; c Ministry of Health and Medical Education, Tehran, Iran.

**Keywords:** Antioxidant activity, Fruit extract, *Punica granatum*, Lythraceae

## Abstract

Pomegranate (*Punica granatum L.*), Lythraceae, is mainly grown in Mediterranean region. It is one of the major cultivated productions of Iran, which have been used in folk medicine for many centuries. It has been proved that pomegranate has a high antioxidant activity and is effective in the prevention of atherosclerosis. This study compares the antioxidant activity, total phenolic and flavonoid contents of nine different pomegranate cultivars grown in Iran. Aqueous solutions of known Fe^+2^ concentration, vitamin E, vitamin C, gallic acid and catechin were used for calibration. The results showed that Sour summer pulp cultivar had the most antioxidant effect with significant difference with the other cultivar (p < 0.05) which can be introduced as a potent source of natural antioxidants, and the peel of three cultivars (Sweet saveh malas, Sour summer and Black peel) as a suitable source for extraction and purification of phenolic and flavonoid compound. The antioxidant capacity of pomegranate peel extract is 10 times higher than the pulp extract.

## Introduction

Epidemiological studies show that consumption of fruits and vegetables with high phenolic and flavonoid contents are correlated with reduced cardiovascular (1, 2), inflammation (3, 4), cancer mortality (5-8) and some other disease rates (9, 10).

Polyphenolic compounds consist of different phenolic rings, out of which one of the major subgroups of these secondary metabolites are flavonoids. They show some functionality in the plant related to interaction with environment such as plant protection against ultraviolet radiation (11-13) and antimicrobial properties to protect plants against micro organisms (14).

As human consumption aspects, flavonoids are one of the major groups of phytochemicals with high antioxidant activity; they have been interesting subjects for general studies in recent years.

Pomegranate (*Punica granatum *L.), Lythraceae, is mainly grown in Mediterranean regions and is one of the major cultivated productions of Iran. It has been consumed for many centuries or perhaps millenniums as fruit, beverage and food-related product. Pomegranate has been used in Iranian traditional medicine for different therapies. For example, the fruit was effective as diuretic and prokinetic agent and also as liver revival. Some other parts of pomegranate tree were also used in anti-parasite and anti-diarrhea formulations. It was also applied in cosmetic and toiletries because of its styptic properties. Today pomegranate is known as antimicrobial (15-17), antiviral (18, 19) and anticancer (5-8) substance which has led to being the center of attention in many studies.

Both pomegranate pulp and peel contain different kinds of antioxidants (20), including those which have not possibly been well characterized so far. It has been acknowledged that phenolic compounds such as flavonoids and anthocyanins are the major class of effective antioxidants in many fruits and vegetables. In this paper the antioxidant activity, total phenolic and flavonoid contents of 9 different Iranian pomegranate cultivars were studied.

## Experimental

*Chemicals*

All reagents and solvents were purchased from Merck (Darmstadt, Germany) and Sigma (St. Louis, MO) unless otherwise mentioned. All chemicals used in the experiments were of analytical grade.

*Sample preparation*

Nine cultivars of pomegranate were donated from Saveh Agricultural Investigation Center during September 2007. A total of 27 pomegranate fruits (three numbers of each cultivar) were collected and washed three times with distilled water. To prepare pomegranate extract, fresh fruits were peeled and their edible portions (seed coats and juice) were separated. 30 g of pulps and peels were weighted and extracted separately for 4 h by Soxhlet apparatus with acetone, followed by ethyl acetate, methanol and water solvent respectively (four hours for each solvent) to extract different kinds of antioxidant components. The four different extracts of every cultivar were mixed and dried on a water bath. 1 g of pulp and peel extracts were dissolved and diluted with methanol 80% (v/v) to 25 mL. To assay total antioxidant and phenolic content, pulp extracts were diluted 1 : 10 (v/v) where it was 1: 100 (v/v) for peel extracts by 80% methanol. 

*Total flavonoid assay*

Total flavonoid content was measured by the aluminum chloride colorimetric assay (21). An aliquot (1 mL) of extracts or standard solution of catechin (50, 100, 150, 200, 250 and 300 mg/L) was added to 10 mL volumetric flask containing 4 mL of double distilled water. Then 0.3 mL 5% NaNO_2_ was added to the flask and after 5 min, 0.3 mL AlCl_3_ (10%) was also added. At 6^th^ min, 2 mL NaOH (1 M) was added and the total volume was made up to 10 mL with double distilled water. The solution was mixed completely and the absorbance level was measured versus prepared reagent blank at 510 nm. Total flavonoid content was expressed as mg catechin equivalents (CE) per one gram dry extract. The total flavonoid assay was measured three times for each pomegranate extract. 

*Total phenolic assay*

Total phenolics were determined using Folin-Ciocalteu reagent as described by Velioglu *et al*. (22) with slight modifications. The extract (200 μL) was mixed with 1.5 mL of Folin-Ciocalteu reagent (previously diluted 10 times with distilled water) and allowed to stand at room temperature for 5 min. 1.5 mL sodium bicarbonate solution (60 g/L) was added to the mixture and after incubation for 90 min at room temperature, the absorbance level was measured at 750 nm using a UV-Visible spectrophotometer (GBC, Cintra 40). Total phenolics were quantified by calibration curve obtained from measuring the absorbance of the known concentrations of gallic acid standard solutions (25-150 μg/mL in 80% methanol). The results were calculated as gallic acid equivalent (GAE) per one gram dry extract and reported as mean value ± SD.

*Total antioxidant activity*

The FRAP (ferric reducing antioxidant power) assay was described initially by Benzie and Strain (23). It is based on the reduction of a ferric tripyridyl triazine complex to its ferrous blue colored form in the presence of antioxidants. It is a relatively simple method frequently used in the assessment of antioxidant activity of various fruits, vegetables and some biological samples (24). 

Briefly, the FRAP reagent contained 5 mL of TPTZ (2, 4, 6-tripyridyl-s-triazine) solution (10 mmol/L) in HCl (40 mmol/L) plus 5 mL of FeCl_3_ (20 mmol/L) and 50 mL of acetate buffer (0.3 mol/L, pH 3.6). It was freshly prepared and warmed up to 37°C. A volume of 100 μL pulp or peel extract was mixed with 3 mL of FRAP reagent and the absorbance of the reaction mixture was measured at 593 nm after incubation at 37°C for 10 min. To construct calibration curve, five concentrations of FeSO_4_ 7H_2_O (1000, 750, 500, 250, 125 μmol ∕ L) were used and the absorbance were measured as sample solution. The values were expressed as the concentration of antioxidants having a ferric reducing ability equivalent to that of 1 mmol/L FeSO_4_ and also as vitamin E and C equivalent. All the measurements were taken in triplicate and expressed as mean value ± standard deviation (SD). 

*Statistical analysis*

Three replicates of each sample were used for statistical analysis and the values were reported as mean ± SD. Pearson’s correlation and regression analysis were carried out using SPSS statistical program to study the relationship between antioxidant activity and total phenolic and flavonoid content. Data were also subjected to the analysis of variance and mean values were compared by Dunnett T3 post-hoc multi-comparison tests. Differences at p < 0.05 were considered to be significant.

## Results and Discussion

The flavonoid content in the pulp and peel extracts is expressed in terms of catechin equivalent (the standard curve equation: y = 0.005x + 0.1478, r^2^ = 0.9919) ranged from 0.84 ± 0.08 to 2.14 ± 0.11 and 18.61 ± 0.53 to 36.40 ± 1.34 mg catechin equivalents per gram of extract respectively (Tables 1 and 2). Tables 1 and 2 also show the content of phenolic compounds that were measured in terms of gallic acid (the standard curve equation: y = 0.005x - 0.0234, r^2^ = 0.9975). The total phenolic contents in the pulp and peel extracts varied from 11.62 ± 0.63 to 21.03 ± 1.51 and 98.24 ± 4.81 to 226.56 ± 18.98 mg gallic acid equivalents per gram of extract respectively. The flavonoid-phenolic ratio in Tables 1 and 2 is mentioned to show the importance of flavonoids in total phenolic content and its antioxidant activity (25). Antioxidant activity in pulp and peel extracts in terms of vitamin C, vitamin E and Fe^+2^ equivalent are shown in Tables 3 and 4. 

**Table 1:** The total phenolic and flavonoid content of pulp extracts in nine different pomegranate cultivars.

	**Cultivar**	**Total phenolic** **mg GAE/g extract**	**Total flavonoids** **mg CE/g extract**	**Flavonoids/ Phenolics**
**1**	Sweet white peel	19.35 ± 1.07	1.22 ± 0.07	0.063
**2**	Agha mohammad ali	11.62 ± 0.63	1.96 ± 0.14	0.168
**3**	North white peel	14.94 ± 1.60	2.14 ± 0.11	0.148
**4**	Sour white peel	16.92 ± 1.63	0.84 ± 0.08	0.050
**5**	Sweet malas	17.65 ± 1.28	1.11±0.08	0.063
**6**	Sour Summer	21.03 ± 1.51	1.46 ± 0.05	0.069
**7**	Sweet saveh malas	19.22 ± 0.71	0.95 ± 0.06	0.049
**8**	Sweet alac	19.93 ± 0.42	1.34 ± 0.09	0.067
**9**	Black peel	19.06 ± 1.42	1.09 ± 0.08	0.057

**Table 2:** The total phenolic and flavonoid content of peel extracts in nine different pomegranate cultivars.

	**Cultivar**	**Total phenolic** **mg GAE/g extract**	**Total flavonoids** **mg CE/g extract**	**Flavonoids/ Phenolics**
**1**	Sweet white peel	220.10 ± 11.23	25.05 ± 0.56	0.114
**2**	Agha mohammad ali	168.21 ± 13.9	33.52 ± 0.41	0.199
**3**	North white peel	192.72 ± 15.45	26.94 ± 0.48	0.140
**4**	Sour white peel	98.24 ± 4.81	28.30 ± 0.54	0.288
**5**	Sweet malas	121.11 ± 8.69	18.61 ± 0.53	0.154
**6**	Sour Summer	226.56 ± 18.98	35.92 ± 0.84	0.159
**7**	Sweet saveh malas	216.74 ± 19.01	34.71 ± 1.34	0.130
**8**	Sweet alac	184.10 ± 25.07	30.36 ± 2.44	0.165
**9**	Black peel	250.13 ± 33.03	36.40 ± 1.34	0.146

**Table 3:** The total antioxidant activity of pomegranate pulp extract as FRAP value, in comparison with vitamins E and C

	**Cultivar**	**micromol ** **Fe ** ^II^ **/g extract**	**mg Vitamin ** **E/g extract**	**mmol Vitamin ** **C/g extract**
**1**	Sweet white peel	325.697 ± 15.551	81.398 ± 4.396	0.160 ± 0.009
**2**	Agha mohammad ali	279.333 ± 7.684	69.433 ± 2.172	0.136 ± 0.004
**3**	North white peel	343.349 ± 18.311	85.953 ± 5.177	0.169 ± 0.010
**4**	Sour white peel	347.255 ± 22.103	86.961 ± 5.704	0.171 ± 0.011
**5**	Sweet malas	316.938 ± 15.431	79.137 ± 5.246	0.155 ± 0.002
**6**	Sour Summer	467.817 ± 10.818	118.074 ± 3.058	0.234 ± 0.060
**7**	Sweet saveh malas	347.104 ± 14.873	86.922 ± 4.205	0.171 ± 0.008
**8**	Sweet alac	410.349 ± 6.412	103.243 ± 1.813	0.204 ± 0.004
**9**	Black peel	312.052 ± 8.302	77.876 ± 2.347	0.153 ± 0.005

**Table 4:** The total antioxidant activity of pomegranate peel extract as FRAP value, vitamins E and C equivalent

	**Cultivar**		**micromol ** **Fe** ^II^**/g extract**	**mg Vitamin ** **E/g extract**	**mmol Vitamin ** **C/g extract**
**1**	Sweet white peel	4560.331 ± 63.451		1150.363 ± 16.173	2.277 ± 0.033
**2**	Agha mohammad ali	3401.354 ± 118.711		851.237 ± 30.635	1.675 ± 0.062
**3**	North white peel	4608.155 ± 96.655		1162.715 ± 24.821	2.301 ± 0.050
**4**	Sour white peel	4164.748 ± 73.967		1048.051 ± 18.712	2.071 ± 0.038
**5**	Sweet malas	3900.882 ± 433.021		979.798 ± 111.829	1.934 ± 0.225
**6**	Sour Summer	4788.401 ± 248.400		1209.126 ± 64.025	2.395 ± 0.129
**7**	Sweet saveh malas	4313.445 ± 140.326		1086.586 ± 36.223	2.148 ± 0.073
**8**	Sweet alac	3993.205 ± 133.149		1003.871 ± 34.226	1.982 ± 0.069
**9**	Black peel	4607.206 ± 78.405		1162.325 ± 20.131	2.301 ± 0.040

As it can be seen, the highest antioxidant activity and phenolic contents were found in Sour summer cultivar for pulp and peel extracts, but the flavonoid content was not very high in pulp extract of this cultivar (1.46 ± 0.05 mg CE/g). The highest flavonoid content can be seen in North white peel (2.14 ± 0.17 mg CE/g) for pulp extract and in Black peel for peel (36.40 ± 1.34 mg CE/g), but the Sour white peel and the Sweet malas cultivars have the least flavonoid content in pulp and peel extract respectively. Also, based on antioxidant activity and total phenolic and flavonoid content, the peel extracts is more potent (near ten folds) than the pulps, indicating that peel extract has more potential effective compounds. Pearson’s correlation only show significant relationship between FRAP and phenol content in pulp (R^2^ = 0.45, p-value = 0.045). The relationship among FRAP, phenol and flavonoid in peel is not significant, therefore the high antioxidant activity in peel is not depended to the phenolic and flavonoid contents. Li *et al*. (26) in 2006 have reported the antioxidant activity of pomegranate peel in comparison with the pulp extract. Their report show similar ratio antioxidant activity for peel-pulp extract. Hajimahmoodi *et al*. (27) in 2008 reported the antioxidant activity of ten Persian pomegranate cultivar hydro-extracts. Based on their findings, the highest FRAP value for pulp and peel was observed in Sour alac (143.9 ± 4.2 micromol/g hydro extract) and Sweet white peel (2826.66 ± 252.75 micromol/g hydro extract) respectively. In comparison with present pomegranate study, hydro extract had less antioxidant activity (about half) compared to pomegranate mixture solvent extract indicating that the solvent mixture is more powerful than the hydro extract for effective compound extraction. Surveswaran *et al*. (28) in 2007 have reported a systematic evaluation of natural phenolic antioxidants from 133 medicinal plants in India. In their report, the pomegranate seed and the pericarp show FRAP value between 0.94 and 19.22 μmol trolox per gram dry weight. As it can be seen in Tables 3 and 4, Sour summer cultivar with 118.074 mg or 274.132 μmol trolox per gram extract has exhibited the highest antioxidant activity. Based on Li *et al*. report (26), using the mixture of solvents was more effective than one solvent extraction to extract antioxidant compounds. The total phenolic content in pulp and peel was estimated 24.4 ± 2.7 and 17.2 ± 3.3 (mg tannic acid/g extract) and the flavonoid content was 249.4 ± 17.2 and 59.1 ± 4.8 (mg rutin/g extract). As it can be seen in Table 1, the range of phenolic and flavonoid contents in pomegranate pulp extract was 11.62 ± 0.63 to 21.03 ± 1.51 mg gallic acid and 0.84 ± 0.08 to 2.14 ± 0.11 mg catechin per gram of extract respectively. It is obvious that the flavonoid contents in their reports were more than the present study. Stangeland *et al*. in 2009 (29) evaluated the total antioxidant activity in 35 Ugandan fruits and vegetables. Among the tested samples, antioxidant activity of pomegranate was 5.11 ± 0.61 mmol Fe^2+^ per 100 g fresh weight. 

In this report, antioxidant activity in Sour summer cultivar with FRAP value equivalent to 4.678 ± 1.818 mmol Fe^2+^ per 100 g fresh weight is comparable with the measure reported in Stangeland›s study. To sum up, Sour summer cultivar is a potent source of natural antioxidants, phenolic and flavonoid content for beverage industry and the peel of three cultivars (Sweet saveh malas, Sour summer and Black peel) are suitable sources of phenolic and flavonoid compound. This study is the most comprehensive comparison among different pomegranate cultivars in the basis of antioxidant activity, phenolic and flavonoid contents. Further studies on the effective antioxidants contained in these fruit fractions and the mechanisms by which they protect against disease development are highly recommended.
